# Association between pubertal development stages and body adiposity in children and adolescents

**DOI:** 10.1186/s12955-020-01342-y

**Published:** 2020-04-06

**Authors:** Fernando Adami, Jucemar Benedet, Livia Akemi Ramos Takahashi, Adair da Silva Lopes, Laércio da Silva Paiva, Francisco de Assis Guedes de Vasconcelos

**Affiliations:** 1 Bolsista do Conselho Nacional de Desenvolvimento Científico e Desenvolvimento Tecnológico – CNPq, Brasília, Brazil; 2 Laboratório de Epidemiologia e Análise de Dados, Departamento de Saúde da Coletividade, Centro Universitário Saúde ABC, Avenida Príncipe de Gales, 821 – Vila Príncipe de Gales, Santo André, SP CEP: 09060-650 Brazil; 3 Instituto Brasileiro de Ensino e Pesquisa Aplicada – IBESPA, Sao Jose, Brazil; 4 Departamento de Educação Física, Programa de Pós Graduação em Educação Física - Centro de Desportos - Universidade Federal de Santa Catarina – UFSC, Campus Universitário – Trindade, Florianópolis, SC CEP: 88040.900 Brazil; 5 Bolsista da Fundação de Amparo à Pesquisa do Estado de São Paulo – FAPESP, São Paulo, Brazil; 6 Departamento de Nutrição, Programa de Pós-graduação em Nutrição do Centro de Ciências da Saúde – Universidade Federal de Santa Catarina – UFSC, Centro de Ciências da Saúde, Campus Universitário – Trindade, Florianópolis, SC CEP: 88040-900 Brazil

**Keywords:** Puberty, Pubertal development stages, Overweight, Obesity, Child, Adolescent

## Abstract

**Background:**

The study aimed to analyze the association between pubertal development stages and adiposity in children and adolescents.

**Methods:**

Cross-sectional study conducted in 2007 in Florianópolis, Brazil, with 2339 schoolchildren 32 aged 8 to 14 years old (1107 males). The outcome (adiposity) was evaluated using Z score and 33 percentilee values > = 85 of four skinfolds (triceps, subscapular, suprailiac and calf) and waist 34 circumference. Total body adiposity (Z score of the sum of skinfolds), central adiposity (Z score 35 of waist circumference) and peripheral adiposity (Z scores of triceps and calf skinfolds) were 36 estimated. Pubertal development stages was self-assessed according to Tanner stages of development. Thirty-seven Children and adolescents were classified according to tertiles of age for each pubertal development stages 38 stage - early, normal and late. Statistical analysis was performed using univariate and 39 multivariate Poisson regression models.

**Results:**

Children and adolescent from both sexes with early pubertal development stages presented higher prevalence of central adiposity (waist circumference), with adjusted prevalence ratio (CI 95%) of 2.21 (1.12; 4.35) for males and 2.18 (1.04; 4.57) for females (reference group: normal pubertal development stages). Among females, there was a reduction in the prevalence of excess adiposity with decreased occurrence of early pubertal development stages. There was a strong relationship between adiposity and pubertal development stages.

**Conclusions:**

Excess adiposity was higher in both sexes for those with early pubertal development stages.

## Plain English summary

To investigate the association of pubertal development stages and body adiposity is relevant, given the importance of fat mass quantity and distribution to health and development and the close relationship between pubertal development stages and body composition of children and adolescents. Cross-sectional study conducted in Florianópolis, Brazil, with schoolchildren aged 8 to 14 years old. Children and adolescent from both sexes with early pubertal development stages presented higher prevalence of central adiposity (waist circumference). Among females, there was a reduction in the prevalence of excess adiposity with decreased occurrence of early pubertal development stages. There was a strong relationship between adiposity and pubertal development stages. Excess adiposity was higher in both sexes for those with early pubertal development stages. These findings have important public health implications given that childhood obesity can be a predictor of chronic diseases in adulthood.

## Introduction

Puberty is a critical period for changes in body composition due to rapid physical growth and significant alterations in the amount and distribution of fat and fat-free mass. Understanding the timing and variability of these changes may provide important information on the health of children and adolescents and on the reasons some individuals reach adulthood at higher risks of obesity, cardiovascular disease, dyslipidemia, hypertension, diabetes, psychosocial disorders and other associated morbidities [1–3]. There is evidence that anthropometric changes occurring during childhood and adolescence can predict body composition and health in adulthood [4, 5].

Body adiposity is a result of the amount of fat accumulated in various body sites. The World Health Organization (WHO) has recommended that body adiposity be assessed by skinfolds, especially in adolescence [6]. In this period, due to pubertal development stages, body changes occur differently according to sex. Male children and adolescents tend to gain larger amounts of lean mass, while females accumulate more body fat [7, 8]. Differences are also observed with regard to body fat distribution; while females tend to accumulate fat peripherally and in the hips, males tend to accumulate fat in the waist region [9–11].

Studies conducted with female children and adolescents have shown that early maturation may be positively associated with body adiposity [12–15]. In males, results are conflicting, however, there are studies indicating increased risk of obesity in boys with early pubertal development stages [16, 17]. Regardless of sex, changes in lean and fat mass tend to last throughout puberty, influencing body characteristics in adulthood [18].

To investigate the association of pubertal development stages and body adiposity is relevant, given the importance of fat mass quantity and distribution to health and development and the close relationship between pubertal development stages and body composition of children and adolescents. Thus, we conducted this study to better understand the real association between weight gain and the physiological process of pubertal development stages. This issue is important for public health due to the consequent comorbidities associated with obesity. Thus, if another study suggests that early pubertal development stages may predispose to future obesity, an alert should be dismissed for the medical community to create measures to regulate children’s pubertal development stages and appropriate weight gain for the age group.

Therefore, the objective of the study was to analyze the association between pubertal development stages and adiposity in children and adolescents, aged 8–14 years old, from Florianópolis, SC, Brazil.

## Methods

This was a cross-sectional study performed in the city of Florianópolis, Santa Catarina state, South of Brazil, in 2007, from April to October. The city has an infant mortality rate of 10.8 per 1000 live births, life expectancy of 87.3 years, and Human Development Index (HDI) of 0.847, all above the national average [19]. The closest relatives, carers or guardians had to consent writing children’s participation in the study according with a term Informed Consent (IC). The study was approved by the Ethics Committee of the Federal University of Santa Catarina (n° 028/06). In addition to this, all methods were performed in accordance with the relevant guidelines and regulations.

The sampling procedure is described in detail elsewhere [20–23]. Briefly, for sample size calculation, the following parameters were assumed: excess weight prevalence of 22.1% for children aged 7–10 years [24] and 12.6% for those aged 11–14 years [25]; acceptable error of 3 percentage points; two-tailed test; confidence level of 95%; design effect of 1.5; and an addition of 10% for losses. Subjects were excluded if the parents or primary caregiver did not sign the written informed consent. The sampling procedure was probabilistic, stratified by clusters and performed in two stages (school and children). Schools were grouped in four strata, according to the geographical area (center/continent or beaches) and school type (public or private). In the first stage, schools were randomly selected from each stratum. From a total of 87 schools (33 private and 54 public), 17 schools were selected (6 private and 11 public). In the second stage, school children were randomly selected according to age. For the present study, 7 year old children (*n* = 421) were excluded, as there was no information on pubertal development stages. The final sample consisted of 2412 school children aged 8 to 14 years (1144 males, 47.4%).

Anthropometric measurements were performed according to WHO guidelines [6], proposed by Lohman et al. [26] The team responsible for data collection included 10 people previously trained in a workshop held between September 2006 and March 2007, which consisted of theoretical and practical lessons on anthropometric measurements. Afterwards, there was a pilot study in two schools (one public and one private) to assess intra- and inter- observer errors in anthropometric measures. Thus the results of interobserver technical errors of measurement and reliability were similar to intraobserver assessment. This suggests that the standardization process of anthropometric measurements was carried out with success [27]. Both schools were excluded from the sampling of the study.

Outcome variables were analyzed in three different dimensions: (a) sum (Σ) of the measure of four skinfold thickness (tricipital, subscapular, suprailiac and calf); b) measures of triceps or calf skinfold thickness only; c) waist circumference values. All these variables were initially analyzed as quantitative variables, using Z score values (Σ skinfolds Z score, tricipital skinfold Z score, calf skinfold Z score, and waist circumference Z score), based on the mean and standard deviation of each anthropometric measure according to sex and age (divided in categories of 6 months). The outcomes were also analyzed as qualitative variables, using a cutoff point at the 85th percentilee to allocate people into two groups: those with excess body fat (percentilee > = 85th) and those without excess body fat (percentilee <85th). In summary, the study outcomes comprise: a) total adiposity (Σ skinfold Z score or percentilee > = 85); b) central adiposity (waist circumference Z score or percentilee > = 85); c) peripheral adiposity (triceps skinfold or calf skinfold Z score or percentilee > = 85). It is important to note that there is no consensus in the current scientific literature about the most adequate cutoff point to classify obesity based on body fat. However, many studies have been using the cutoff point of 85th percentilee to define excess body fat [28–31].

Pubertal development stages was determined based on the stages of development [32] Children and adolescents received instructions individually by a research team member in a private environment and were asked to perform a self-evaluation. Tertiles of age (in decimals) for each of the 5 development stages were estimated for both sexes [33]. For each stage, school children were divided in early pubertal development stages (below first tertile of age), late pubertal development stages (second tertile or more) and normal pubertal development stages or reference group (between the first and the second tertile).

The study covariables were collected and analyzed as follows. Birth weight was reported by the parent or primary caregiver, who was asked to check the child’s health record. The subjects were classified into low (< 2.500 g), normal (≥ 2.500 g - 3.999 g) and high birth weight (≥ 4.000 g) [6]. The nutritional status of the mother was evaluated by BMI, using self-reported weight and height. Excess body weight classification (≥ 25 kg/m^2^) followed WHO recommendation [34]. Information on the mode of commuting to school was collected by an illustrated questionnaire [21] and subjects were classified as active (walking and biking) or inactive (car, bus, passenger in motorcycle or bicycle). Further information (name, date of birth, school grade and type of school) were obtained in documents provided by the school. The type of school refers to public or private.

Quantitative variables were described as median, 25th and 75th percentilees, given the non-normal distribution of anthropometric data (Shapiro-Wilk test, *p* < 0.05). Qualitative variables were described by relative frequencies (%). Quantitative variables were compared between sexes using Mann-Whitney test. Kruskal-Wallis test was used to compare outcomes according to pubertal development stages (early, normal or late). The associations among qualitative variables were assessed by Rao-Scott test. The relation between the independent variables and excess adiposity (0-absence; 1-presence) was tested with univariate and multivariate Poisson regression models, using robust variance and stepwise forward strategy (multivariate model). Stata 11.0® was used in all statistical analysis, including the command svy to allow for sampling weights and stratification [35, 36].

## Results

Seventy-three individuals (3.03%) were excluded for the following reasons: height Z scores greater than 5 (1 girl) and missing or inconsistent data on pubertal development stages (36 males and 36 females). The final sample comprised 2339 children and adolescents aged 8–14 years old. No significant differences were found in the prevalence of total, central or peripheral excess adiposity or in the study covariables between subjects excluded from the analysis in comparison to the final sample. Male children and adolescent accounted for 47.7% of the study sample. Age, adiposity Z scores and excess adiposity were similar between sexes. Differences were observed in relation to birth weight (*p* < 0.001), with higher proportion of males born with high weight values (14.4% vs 8.1% in females) (Table 1).

**Table 1:** Characteristics of children and adolescents aged 8 to 14 years old in Florianópolis, SC, South of Brazil, 2007

Study variables	Males (47.7%)	Females (52.3%)	p^b^
	Median (p25; p75)^a^		
Age (years)	11 (10; 13)	12 (10; 13)	0.529
Σ skinfold Z score^c^	−0.36 (− 0.73; 0.52)	− 0.22 (− 0.72; 0.50)	0.501
Waist circumference Z score	−0.24 (− 0.71; 0.53)	−0.17 (− 0.67; 0.50)	0.618
Tricipital skinfold Z score	−0.24 (− 0.76; 0.60)	−0.17 (− 0.74; 0.54)	0.550
Calf skinfold Z score	−0.29 (− 0.75; 0.61)	−0.18 (− 0.74; 0.52)	0.555
	%		
Σ skinfold Z score - percentile > = 85	9.7	8.9	0.436
Waist circumference Z score - percentile > = 85	8.8	7.6	0.257
Tricipital skinfold Z score - percentile > = 85	10.2	9.3	0.414
Calf skinfold Z score - percentile > = 85	9,5	8,5	0,468
Sexual maturation classification			0.937
Early	33.1	32.5	
Late	32.2	32.7	
Maternalr excess body weight (BMI)	31.0	33.4	0.313
Birth weight (grams)			< 0.001
< 2.500	5.8	8.3	
> 4.000	14.4	8.1	
Active commuting	43.6	45.0	0.558
School type			0.975
Public	65.4	65.5	
Private	34.6	34.5	

^a^p25 e p75: percentiles 25 and 75, respectively

^b^Mann-Whitney test (qualitative variables) and Rao-Scott (quantitative variables)

^c^Sum of tricipital, subescapular, suprailiac and calf skinfold thickness

Comparing male children and adolescents, there were no statistically significant differences in adiposity and excess adiposity variables according to pubertal development stages (early, normal, late) (Table 2). It is noteworthy, however, the upward trend of the waist circumference Z- score related to early pubertal development stages (P for trend = 0.035). In female children and adolescents, there was a statistically significant association of adiposity (central and peripheral) and excess adiposity (Σ skinfolds) with pubertal development stages, with the exception of triceps skinfold Z score - percentilee < 85 (*p* = 0.105). Thus, an increasing trend of adiposity values and excess body fat prevalence with early age of pubertal development stages was observed (trend *p* < 0.001 for all variables).

**Table 2:** Hypothesis test and descriptive values of adiposity measurements according to pubertal development stages in children and adolescents (8–14 years old), Florianópolis, SC, South of Brazil, 2007

Variables	Pubertal development stages					Pubertal development stages				
	Early	Normal	Late	p^b^	p^c^	Early	Normal	Late	p^b^	p^c^
	Males					Females				
	Median (p25; p75)^a^					Median (p25; p75)^a^				
Σ skinfold Z score^d^	−0.39 (−0.77; 0.61)	−0.29 (−0.74; 0.62)	−0.39 (−0.69; 0.36)	0.752	0.503	−0.04 (−0.50; 0.78)	−0.25 (− 0.73; 0.45)	−0.43 (− 0.88; 0.19)	< 0.001	< 0.001
Waist circumference Z score	− 0.16 (− 0.67; 0.59)	−0.22 (− 0.72; 0.54)	−0.29 (− 0.75; 0.31)	0.106	0.035	0.08 (− 0.42; 0.77)	−0.17 (− 0.67; 0.41)	−0.45 (− 0.84; 0.22)	< 0.001	< 0.001
Tricipital skinfold Z score	− 0.33 (− 0.84; 0.64)	−0.16 (− 0.76; 0.59)	−0.27 (− 0.69; 0.51)	0.416	0.203	0.08 (− 0.54; 0.81)	−0.20 (− 0.75; 0.53)	−0.43 (− 0.85; 0.23)	< 0.001	< 0.001
Calf skinfold Z score	− 0.32 (− 0.78; 0.68)	−0.25 (− 0.74; 0.58)	−0.25 (− 0.74; 0.54)	0.838	0.562	− 0.03 (− 0.53; 0.67)	−0.16 (− 0.70; 0.57)	−0.39 (− 0.89; 0.33)	< 0.001	< 0.001
	%					%				
Σ skinfold Z score - percentile > = 85	9.4	7.9	12.2	0.105	0.485	13.8	7.9	5.4	0.014	< 0.001
Waist circumference Z score - percentile > = 85	11.2	6.1	8.3	0.119	0.214	11.4	6.5	5.1	0.034	< 0.001
Tricipital skinfold Z score - percentile > = 85	9.6	9.7	11.6	0.661	0.746	12.3	8.3	6.1	0.105	< 0.001
Calf skinfold Z score - percentile > = 85	8.8	9.3	8.7	0.921	0.949	11.9	6.2	7.1	0.016	< 0.001

^a^p25 and p75: Percentiles 25 and 75

^b^Kruskal-Wallis test (quantitative variables) and Rao-Scott test (qualitative variables)

^c^P for trend

^d^Sum of tricipital, subescapular, suprailiac and calf skinfold thickness

In the adjusted model (Tables 3 and 4), children and adolescent from both sexes with early pubertal development stages presented higher prevalence of central adiposity (waist circumference), with adjusted prevalence ratio (CI 95%) of 2.21 (1.12; 4.35) for males and 2.18 (1.04; 4.57) for females (reference group: normal pubertal development stages). Among females, there was a reduction in the prevalence of excess adiposity with decreased occurrence of early pubertal development stages, all the prevalence ratio values being lower than 1 for those who experienced late pubertal development stages.

**Table 3:** Estimates from multivariate Poisson regression models for excess adiposity acoording to pubertal development stages in males (8–14 years old), Florianópolis, SC, South of Brazil, 2007

Pubertal development stages in males	Σ skinfold^c^ Z score - percentile > = 85	Waist circumference Z score - percentile > = 85	Tricipital skinfold Z score - percentile > = 85	Calf skinfold Z score - percentile > = 85
Adjusted prevalence ratio (CI 95%)^ab^				
Early	1.38 (0.87; 2,20)	2.21 (1.12; 4.35)	1.10 (0.64; 1.90)	1.0 (0.65; 1.58)
Normal	1	1	1	1
Late	1.86 (0.93; 3.73)	1.41 (0.59; 3.34)	1.42 (0.56; 3.57)	0.99 (0.52; 1.86)

^a^Confidence interval of 95%

^b^Adjusted for maternal BMI, active commuting, age, birth weight, school type and interaction between maternal BMI and school type using Poisson regression models

^c^Sum of tricipital, subescapular, suprailiac and calf skinfold thickness

**Table 4:** Estimates from multivariate Poisson regression models for excess adiposity acoording to pubertal development stages in females (8–14 years old), Florianópolis, SC, South of Brazil, 2007

Pubertal development stages in females	Σ skinfold^c^ Z score - percentile > = 85	Waist circumference Z score - percentile > = 85	Tricipital skinfold Z score - percentile > = 85	Calf skinfold Z score - percentile > = 85
Adjusted prevalence ratio (CI 95%)^ab^				
Early	1.92 (0.89; 4,13)	2.18 (1.04; 4.57)	1.74 (0.88; 3.44)	1.96 (1.00; 3.85)
Normal	1	1	1	1
Late	0.58 (0.28; 1.21)	0.61 (0.29; 1.26)	0.58 (0.33; 1.02)	0.91 (0.42; 1.97)

^a^Confidence interval of 95%

^b^Adjusted for maternal BMI, active commuting, age, birth weight, school type and interaction between maternal BMI and school type using Poisson regression models

^c^Sum of tricipital, subescapular, suprailiac and calf skinfold thickness

## Discussion

The results of the study showed that early pubertal development stages is associated with increased waist circumference in children and adolescents in both sexes. This indicator of central adiposity excess, if assessed in early stages of life, is an important predictor of morbidities in adult life [37], especially type 2 diabetes, coronary heart disease, stroke, sleep apnea, hypertension, dyslipidemia, insulin resistance, inflammation and some cancers [5].

Other studies point to a similar trend of increased waist circumference values in children and adolescent boys with early pubertal development stages [38, 39]. Nevertheless, it should be emphasized that, regardless of pubertal development stages, there is evidence that boys may present higher fat concentrations in the waist area since prepuberal period (5–7 years) [9, 11]. Puberty and maturational events can increase the concentration of fat in the central region, leading to a more android shape in boys [10], given that body fat accumulates secondarily to pubertal development. Endocrine changes present in the pubertal process would be responsible for weight gain. It is known that testosterone levels are low and estradiol levels are high in obese adolescents [40]. This inverse relationship between these hormones would be due to the high level of aromatase in cases of high body weight.

Since estradiol is related to the accumulation of fat and testosterone to the development of hair, its altered levels during puberty would explain why there are studies that associate obesity in children with the late appearance of pubic hair. However, the association among body fat, age and puberty can be non-linear as late puberty can be found both in boys with low fat and in obese boys. Longitudinal studies are needed to answer these questions [41].

Among children and adolescent girls, in the adjusted model, the sum of skinfold thickness was not associated with early pubertal development stages. These findings are similar to Ridder et al. [42], in which adiposity, also assessed by the sum of skinfolds, was not positively associated with early pubertal development stages. On the other hand, in this study, children with early pubertal development stages had excess central adiposity. Ibanez and colleagues [15] found that early pubertal development stages was associated with adiposity in a cross-sectional study with female adolescents. In this study, measures of total body fat and body fat distribution (waist circumference, waist/hip ratio, central abdominal fat) were significantly higher among girls with early pubertal development stages. This same trend was described by Bratberg et al. [14] in a study involving adolescents from 14.2 to 18.2 years of age, in which a larger waist circumference was associated with excess weight and early pubertal development stages.

Biologically, puberty in females is associated with an increase in the amount of fat mass as a consequence of increased blood concentrations of estradiol [18] Furthermore, higher concentrations of insulin, growth factors like IGF-1 [43, 44], and leptin may also favor increases in adiposity [45]. In puberty, particularly in its late phase, leptin levels tend to decrease in males and increase in females, which can contribute to greater fat mass among females [46, 47]. Thus, it is expected that the early onset of puberty in females is related to an increase in the amount of adipose tissue. The results of this study reinforce this trend, given that higher values of adiposity indicators were found among children and adolescents with early pubertal development stages.

The increase in central adiposity in both girls and boys does not seem to be an isolated trend. There is evidence that abdominal obesity is growing faster than general obesity (assessed by BMI) among adolescents. This trend was observed in Spain where waist circumference measures increased, regardless of changes in BMI in both sexes, in adolescents aged 14 years old between 1995 and 2002 [48]. The same trend was observed in waist circumference of British children and adolescents, which increased faster than BMI in the last 10–20 years (1977–1987-1997), especially in females [49].

Although there is some evidence in the literature, the hypotheses to explain the relationship between pubertal development stages and adiposity are still debatable and inconclusive. In this context, regardless of the endocrine aspects of puberty, the role of genetic factors, ethnic and environmental factors cannot be ruled out. Environmental issues have been widely discussed and changes in food consumption and physical activity might play a role in this phenomenon [13, 50]. In fact, there is evidence that a positive energy balance in the long term can affect both the levels of adiposity and the timing of pubertal development stages, causing these trends to be coincident [51].

The determination of early pubertal development stages, although not standardized in the literature, was similar to that used by other authors [16, 33]. A limitation of the study may be related to the use of self-assessment of pubertal development stages technique. However, this procedure on field research has been validated in studies with Brazilian adolescents [40, 52]. The results showed a good correlation (r = 0.80) between the measurements from self-assessment and from experienced personnel. Furthermore, the low sample loss and the adjustment for important predictors of excess body fat decrease the possibility of selection bias, information bias and confounding. This factor, together with the population-based characteristic of our research, increases internal and external validity of our findings.

Thus these new findings what children and adolescents from both sexes with early pubertal development stages presented a higher prevalence of central adiposity (waist circumference). To investigate the association of pubertal development stages and body adiposity is relevant, given the importance of fat mass quantity and distribution to health and development and the close relationship between pubertal development stages and body composition of children and adolescents. These findings have important public health implications given that childhood obesity can be a predictor of chronic diseases in adulthood.

## Conclusion

In summary, our findings support the existence of a strong relationship between adiposity and pubertal development stages. In the adjusted model, adiposity excess, as indicated by waist circumference, was more common in children and adolescents with early pubertal development stages for both sexes. Also, not having an early pubertal development stages proved to be a protective factor for excess body fat in females. It is recommended to observe the pubertal development stages, in addition to chronological age and sex, in the assessment of nutritional status during puberty. In particular, the association between early pubertal development stages and excess body fat is relevant to public health, given the predictive ability of obesity in childhood and adolescence to chronic diseases and obesity in adulthood.

By adding these findings to the knowledge of doctors and health agents, it is possible to create an alert regarding the harmful effects of allowing early maturation to occur, since it is related to weight gain and obesity. This would influence the creation of screening measures and the consequent treatment of precocious puberty. In addition to reinforcing measures and research aimed at delaying pubertal development stages.

## Data Availability

All relevant search data is in the article.

## Abbreviations

IC: Informed consent
HDI: Human Development Index
WHO: World Health Organization
95%CI: 95% confidence interval
